# 

**Published:** 1921-01-01

**Authors:** 


### For the Babies.

The National Association for the Prevention of Infant Mortality will be holding its second English-speaking Congress on Infant Welfare in London on July 5, 6, and 7, 1921. The following are among the subjects to be discussed: Residential Provision for Mothers and Babies; Inheritance and Environment as Factors in Racial Health; The Supply of Milk: its Physiological and Economic Aspects. Further particulars may be obtained from the Secretary at 4 Tavistock Square, London, W.C. 1.

### Gifts and Bequests.

IN answer to the Mayor's Appeal Fund on behalf of the Beckett Hospital and Dispensary, Barnsley, a sum of £21,706 has been received and promised.

CROYDON HOSPITAL has recently benefited to the extent of over £1,000 as the result of the winding-up of a number of local war funds, the remaining credit balances of which having been sent to that institution.

THE Hon. Treasurer of the Royal Southern Hospital, Liverpool, had a pleasant surprise on Christmas morning, when notification was received that under the will of the late David Martin Currie, the hospital had benefited to the extent of £5,000 in 3½ per cent. New Zealand stock.

THE Alexandra Hospital, Queen Square, has been left £100 by Lady Camilla Fortescue.

UNDER the will of the late Mr. Walter Pateshall Jones, of Bournemouth, who died on May 24, 1920, the Royal Victoria Hospital, Bournemouth, is bequeathed a legacy of £2,000, free of duty. The hospital has also received payment of a legacy of £200, less duty, under the will of the late Mrs. S. S. B. Burton, of Branksome Park, Bournemouth, who died on May 15, 1920, and of a legacy of £100, free of duty, bequeathed by the late Mr. D. W. Preston, of Bournemouth, who was for many years a member of the Board of Management of the hospital.

### Matrons' and Sisters' Appointments.

Borough Isolation Hospital, Dover.—Miss M. Savery has been appointed matron. She was trained at Burnley Union Infirmary and Leeds City Hospital, has been sister at Bagthorpe Infirmary, Nottingham, night sister at the Royal Hospital for Children, Glasgow, sister at the Isolation Hospital, East Ham, sister at the Queen's Hospital for Children, Hackney, home sister and assistant matron at the Home of Recovery, Golders Green, and tutor-sister and third assistant matron at the Whipps Cross Hospital, Leytonstone.

Christie Hospital, Manchester.—Miss Maria Hunstone has been appointed sister. She was trained at the Royal Hospital, Sheffield, where she later held the post of sister of the women's medical ward, the women's surgical ward, and is at present home sister. Between these appointments she was on active service in India and Mesopotamia.

Royal Albert Edward Infirmary, Wigan.—Miss Amy H. Wilford has been appointed matron. She was trained at the Royal Infirmary, Sheffield, where she was later successively theatre sister, ward sister, and night superintendent. She is at present assistant matron of the Royal Albert Edward Infirmary, Wigan.

Children's Hospital, Southampton.—Miss Ellen Ede has been appointed sister. She was trained at Camberwell Infirmary, and later was staff nurse there.

The India Office announces that the following ladies have been appointed to Queen Alexandra's Military Nursing Service for India in the grade of staff nurse (temporary): Miss F. M. Allison, Miss M. Carpenter, Miss M. E. Knight, Mrs. E. Law, and Miss M. T. T. Thomson.

### Medical Appointments.

Dr. Michele Sicca has been appointed house surgeon to the Italian Hospital, and took up his duties on December 1.

Dr. D. W. Cowin, of Kendal, Westmorland, has been appointed schools dental surgeon under the Barry (Glamorgan) Education Committee.

Viscountess Hambleden and the Hon. Mrs. Anthony Henley have been appointed members of King's College Hospital Committee of Management.

At St. Thomas's Hospital Medical School the University Scholarship, 1920 (value £100), has been awarded to Mr. C. V. Patrick, of Rugby, and Caius College, Cambridge.

Mr. H. Cuthbert Hopkyns, M.A. (Oxon), of the secretarial department, St. Thomas's Hospital, London, has been appointed to succeed Mr. A. Stanley Brunt as Secretary-Superintendent of the North Ormesby Hospital, Middlesbrough, and is expected to take up his duties in January next.

### The Book Market.

The receipt of the following is acknowledged:—

E. and S. Livingstone, 17 Teviot Place, Edinburgh: "Surgical Anatomy." By C. R. Whittaker, F.R.C.S.E., F.R.S.E. Second edition, Part II. (Catechism Series). 1s. 9d.

H. K. Lewis and Co., Ltd.: "Carter's Elements of Practical Medicine." By A. G. Gibson. Price 16s. net.

John Wright and Sons, Ltd., Bristol: "Injuries of the Peripheral Nerves." By H. S. Souttar, C.B.E., and E. W. Twining. 18s. 6d. net.

Manchester University Press and Longmans, Green and Co.: "Feeblemindedness in Children of School Age." By C. Paget Lapage, M.D., M.R.C.P. Second edition. 10s. 6d. net.

Henry Frowde and Hodder and Stoughton: "Text-Book of Pharmacology and Medical Treatment for Nurses." By J. M. Fortescue-Brickdale. "Surgical Aspects of Dysentery." By Z. Cope, B.A., M.D., M.S., F.R.C.S. 12s. 6d. net.

J. Bale, Sons & Danielsson, Ltd.: "Pocket First-Aid Series," Nos. 1 to 6. By Col. R. J. Blackham. 6d. net each. "Text-book of Tracheo-Bronchoscopy." By D. M. Mann. 31s. 6d. net. "The Medical Examination of Airmen." By Dr. Maublanc and Dr. Ratié. 5s. net.

Cambridge University Press: "Industrial Colonies and Village Settlements for the Consumptive." By Sir German Woodhead and P. C. Varrier-Jones. Paper covers, 9s. net; cloth, 10s. 6d. net.

C. Griffin & Co., Ltd.: "Centenary Volume of Chas. Griffin & Co., Ltd."

J. B. Lippincott Co.: "Normal Histology." By G. A. Pursol, M.D., Sc.D. Twelfth Edition. 21s. net. "Genito-Urinary Surgery and Venereal Diseases." By Martin, Thomas, and Moorhead. 35s. net. "Accessory Sinuses of the Nose." By R. H. Skillern, M.D. 30s. net. "International Clinics." 42s. Set of 4 vols. "Essentials of Medicine." By C. Phillips Emerson, M.D. 12s. 6d. net. "First Aid in Emergencies." By E. L. Eliason, M.D. Third Edition. 7s. 6d. net. "A Nurse's Handbook of Obstetrics." By J. B. Cooke, M.D. Ninth Edition. 12s. 6d. net. "Care and Feeding of Infants and Children." By W. Reeve Ramsey, M.D. Second Edition. 10s. 6d. net. "Simplified Infant Feeding." By R. H. Dennett, B.S., M.D. Second Edition. 21s. net.

Oliver and Boyd, Edinburgh: "Functional Mental Illnesses." By R. G. Rows, M.D., and David Orr, M.D. 3s. 6d. net.

Williams and Norgate: "Some Conclusions on Cancer." By Charles Creighton, M.D. 42s. net.

